# MTMR14 Alleviates Chronic Obstructive Pulmonary Disease as a Regulator in Inflammation and Emphysema

**DOI:** 10.1155/2022/9300269

**Published:** 2022-01-07

**Authors:** Yiya Gu, Jinkun Chen, Qian Huang, Yuan Zhan, Ting Wang, Jixing Wu, Jianping Zhao, Zhilin Zeng, Yongman Lv, Chengfeng Xiao, Jungang Xie

**Affiliations:** ^1^Department of Respiratory and Critical Care Medicine, National Clinical Research Center of Respiratory Disease, Key Laboratory of Pulmonary Diseases of Health Ministry, Tongji Hospital, Tongji Medical College, Huazhong University of Science and Technology, Wuhan, Hubei 430030, China; ^2^Department of Science, Western University, 1151 Richmond Street, London, Ontario, Canada N6A 3K7; ^3^Department and Institute of Infectious Diseases, Tongji Hospital, Tongji Medical College, Huazhong University of Science and Technology, Wuhan, Hubei 430030, China; ^4^Health Management Center, Tongji Hospital, Tongji Medical College, Huazhong University of Science and Technology, Wuhan, Hubei 430030, China; ^5^Department of Biology, Queen's University, Kingston, Ontario, Canada K7L 3N6

## Abstract

Extensive inflammation and apoptosis in structural cells of the lung are responsible for the progression and pathogenesis of chronic obstructive pulmonary disease (COPD). Myotubularin-related protein 14 (MTMR14) has been shown to participate in various biological processes, including apoptosis, inflammation, and autophagy. Nonetheless, the role of MTMR14 in COPD remains elusive. In the present study, we explored the expression of MTMR14 in human lung tissues and investigated the effects of overexpressed MTMR14 on *in vitro* and *in vivo* COPD models. Moreover, one of the possible mechanisms of MTMR14 alleviating COPD was explored based on mitochondrial function and mitophagy homeostasis. The results showed that MTMR14 expression was reduced in COPD patients' lungs in comparison to control subjects. MTMR14 overexpression inhibited cigarette smoke extract-induced inflammation and apoptosis and improved mitochondrial function and mitophagy *in vitro*. Further verification was carried out in COPD model mice. MTMR14 overexpression inhibited lung inflammation and reduced levels of IL-6 and KC in bronchoalveolar lavage fluid, as well as prevented emphysema and a decline in lung function. Furthermore, MTMR14 overexpression improved mitochondrial function and mitophagy to a certain extent. Collectively, our data support the hypothesis that MTMR14 participates in the pathogenesis of COPD. Improving mitochondrial function and mitophagy homeostasis may be one of the mechanisms by which MTMR14 alleviates COPD and may potentially be a novel therapeutic target for COPD.

## 1. Introduction

Chronic obstructive pulmonary disease (COPD) is a chronic inflammatory disorder that has become the fourth most common cause of death worldwide and is associated with a significant social and economic burden. As the first-line defense against noxious insults, the human bronchial epithelium plays a vital role in preventing the onset of COPD [1]. Repeated noxious challenges induce a chaotic immune response and promote the inflammatory process in impaired epithelial cells, leading to tissue destruction and progressive airflow limitation [2].

Inflammation, oxidative stress, and an imbalance between protease and antiprotease are well-known mechanisms responsible for the development of COPD [3]. Recent evidence suggests that the imbalance between replenishment and apoptosis of structural cells may also contribute to lung tissue destruction and the pathogenesis of COPD [4, 5]. The interaction between various pathogenic mechanisms adds complexity to the treatment of this disease [4]. Because it has been challenging to achieve satisfied efficacy with single target therapy, the therapeutic repair of suborganelle function has become the focus of drug development.

Mitochondria, as a widely distributed and functional organelle, have attracted increased attention in targeted therapy for airway diseases [6–8]. Mitochondria are both the main producers of oxygen radicals and a target of their destruction. Mitochondrial dynamics and mitophagy jointly regulate mitochondrial homeostasis and maintain mitochondrial functions. Numerous studies have demonstrated that mitochondrial dysfunction is involved in oxidative stress, inflammation, proliferation, apoptosis, and, of course, metabolism, which are key aspects of the pathophysiology of airway diseases [9].

Myotubularin- (MTM-) related protein 14 (MTMR14) is part of the MTM-related protein family and has been proven to be involved in various diseases [10, 11]. A previous study reported that MTMR14 deficiency resulted in metabolic dysfunction and late-onset inflammation [12]. Consistently, the depletion of MTMR14 accelerated high-fat diet-induced inflammation and lipid accumulation in mice [13]. It has also been found that MTMR14 knockdown inhibits migration and promotes apoptosis in liver cancer cells [14]. Moreover, MTMR14 exerts a negative regulatory effect on autophagy [10, 15, 16]. The aforementioned studies indicate that MTMR14 participates in a multitude of biological processes, such as apoptosis, inflammation, and autophagy. However, the role of MTMR14 in COPD remains unclear. Whether mitophagy, a type of selective autophagy, is also regulated by MTMR14 and further affects mitochondrial function in COPD has not been explored yet.

Our preliminary study indicated that decreased MTMR14 expression may play a crucial role in COPD pathogenesis. To evaluate our hypothesis, a cigarette smoke extract- (CSE-) treated cell model and a COPD mouse model were constructed. We demonstrated that overexpression of MTMR14 reduces inflammation, apoptosis, and the progression of emphysema *in vivo* and *in vitro*. These results indicate that one of the means by which MTM14 plays its biological role may be the regulation of mitochondrial function and mitophagy.

## 2. Methods

### 2.1. Subjects

Participants (age: 40–80 years) who underwent surgery for medical reasons were recruited from the Department of Thoracic Surgery of Tongji Hospital (Wuhan, China). COPD was diagnosed based on the GOLD 2020 criteria. Subjects were categorized into three groups according to pulmonary function and smoking history: the nonsmoker group, the smoker group, and the COPD group. The exclusion criteria were as follows: (1) chronic lung conditions, such as pulmonary fibrosis, asthma, and bronchiectasis; (2) active tuberculosis; (3) renal, hepatic, or cardiac failure; and (4) currently treated with inhaled or oral steroid therapy. Lung tissue specimens (a minimal distance of 5 cm from the lesion) were collected during surgical resection. This study was authorized by the ethics committee of the Tongji Hospital, Huazhong University of Science and Technology in Wuhan, China. Written informed consent was obtained from each participant prior to participation.

### 2.2. Animal Model

Male C57BL/6J mice (aged: 10–12 weeks; weight: 25-30 g) were purchased from GemPharmatech Co., Ltd. (Jiangsu, China; Certificate Number: SCXK (Su) 2018-0008), and housed in a specific pathogen-free animal center at the Tongji Medical College. The mice were fed with sterile acidified water and irradiated food under the light/dark photocycle at 12 : 12 h. The COPD mouse model was established by referring to a previous study [17]. Briefly, mice were either subjected the whole body to room air or cigarette smoke (CS) in a chamber for approximately 3 h per day (10 cigarettes/45 min/time, 4 times per day) and 6 days per week for a total of 2 weeks with Marlboro Red cigarettes (tar: 10 mg, carbon monoxide: 11 mg, nicotine: 0.8 mg; Switzerland). During CS exposure, CO concentration was maintained at 600-1200 ppm and O_2_ concentration >18%. Subsequently, all mice were hosted at room air for an additional 2 weeks. On day 22, 50 *μ*l of MTMR14-Adeno-associated virus (MTMR14-AAV) obtained from OBiO Biotechnology Co. Ltd. (Shanghai) was intratracheally administrated (total amount of titer 2 × 10^11^ vg per mouse, once) to the mouse with the PenWu Device (Bio Jane, Cat. No. BJ-PW-M, Shanghai) to overexpress MTMR14, and an equivalent amount of negative control (NC)-AAV diluted with PBS was applied as vehicle control. From day 31, mice were administered intratracheally with 100 *μ*g elastin in 50 *μ*l saline (Eln) or normal saline (NS) (q.o.d., for a month) and were sacrificed 24 h after the last elastin administration. The mice were divided into four different groups at random: Air+NS, CS+Eln, CS+Eln+NC, or CS+Eln+MTMR14. The animal experiment protocols were authorized by the ethics committee of the Tongji Hospital, Huazhong University of Science and Technology in Wuhan, China (Certificate Number: TJH-202105013).

### 2.3. Pulmonary Function

After the modeling period, the mice were tracheostomized, and the trachea was cannulated. Then, the cannula was connected to a computer-controlled small animal ventilator with a sealed glass cover. It was worth noting that the mice will be given the appropriate anesthesia before these operations. Generally, mice were anesthetized with 1% pentobarbital sodium 10 ml/kg body weight intraperitoneally, and the dose could be adjusted according to the difference of anesthetic tolerances. After endotracheal intubation, the pulmonary function of the mice was measured using the FlexiVent system (SCIREQ, Montreal, Quebec, Canada). The pulmonary function tests, forced expiratory volume at 0.05 s (FEV_0.05s_) and forced vital capacity (FVC), were registered, while the FEV_0.05s_/FVC was calculated.

### 2.4. Histological Analyses

Hematoxylin and eosin (HE) was used to stain the fixed and embedded lung specimens. Light microscopy at the 200x magnification was used to evaluate the degree of peribronchial inflammation and mean linear intercept (MLI) in photomicroscopic images of HE-stained specimens. At least 5 random photomicroscopic images were evaluated per mouse. The grades of inflammation were assessed according to a previously described method [18]. The MLI was calculated to evaluate the degree of emphysema. In brief, cross-reference lines were formed right in the middle of each microscopic field and the total length (*L*) and the number of alveolar septa (NS) were measured. MLI was calculated with the formula: *L*/NS.

### 2.5. Bronchoalveolar Lavage

Bronchoalveolar lavage fluid (BALF) was collected three times (700 *μ*l, 800 *μ*l, and 800 *μ*l, respectively) from each mouse. The first supernatant was collected to assess the level of inflammatory cytokines by ELISA. After resuspension, the total cells were collected through centrifugation to obtain cytospin, and the cell types were classified with Liu's staining (Baso, Zhuhai, China).

### 2.6. Transmission Electron Microscopy

After treatments, 1~3 mm^3^ fresh lung tissues and human bronchial epithelial (HBE) cells were fixated at room temperature in 2.5% glutaraldehyde for 30~60 minutes, then transferred to 4°C for 24 hours. These samples were postfixed in 1% osmium tetroxide and dehydrated using a series of ethanol. Uranyl acetate-lead citrate was used to double-stain the ultrathin sections, and images were taken under the transmission electron microscope ([TEM] HT7800, Hitachi).

### 2.7. TUNEL Staining

The paraffin sections were used to detect the apoptosis of mouse lungs by TUNEL (terminal deoxynucleotidyl transferase dUTP nick-end labeling) staining. The implementation steps were in accordance with the manufacturer's instructions (Servicebio, G1501). Green fluorescence represented positive staining, and DAPI was applied to counterstain nuclei. Be careful to avoid light during the experiment. Images were obtained with a fluorescence microscope.

### 2.8. Cell Culture and Treatment

Human bronchial epithelial cells HBE4-E6/E7 (ATCC, CRL-2078) were preserved in RPMI-1640 medium, which was supplemented with 10% fetal bovine serum, in a humidified incubator at 37°C containing 5% CO_2_. Then, the cells were treated with CSE at different concentrations. In the overexpression experiment, cells were infected with a lentiviral vector (NC or MTMR14-overexpressing), purchased from the OBiO Biotechnology Co. Ltd. (Shanghai), in accordance with the manufacturer's instructions. Western blotting was used to determine the expression level of MTMR14.

### 2.9. Cigarette Smoke Extract Preparation

CSE was acquired as described previously [19]. Briefly, CSE was obtained by collecting smoke from two cigarettes (3R4F, University of Kentucky, Lexington, USA) without filter into a centrifuge tube of 50 ml that contained 20 ml culture medium, while light and wind were avoided with the aid of vacuum extraction. A 0.22 *μ*m filter was used to sterilize the obtained extracts which were deemed as 100% concentrations of CSE.

### 2.10. Immunohistochemistry

Anti-MTMR14 antibody (ABclonal) was used to stain the paraffin-embedded lung tissue sections. The expression of MTMR14 in lung sections was detected using an IgG Streptavidin Biotin Complex kit (Boster). The staining was developed with DAB substrate (Dako, Glostrup, Denmark) and observed using a Nikon Spot image acquisition and processing system (USA).

### 2.11. Western Blot

Lysis buffer containing phosphatase inhibitors was used to extract the total protein from human and mouse lung tissues, as well as HBE cells. The total protein concentration was detected with BCA assay (Bioyear Biotechnology, Wuhan, China). Protein samples were then added with 5× loading buffer, boiled, isolated on sodium dodecyl sulfate-polyacrylamide gels, and relocated to polyvinylidene fluoride membranes. Primary antibodies against MTMR14 (Proteintech, China; ABclonal Biotechnology), Bax, PINK1 (Proteintech, China), Bcl-2 (Boster, Wuhan, China), Cleaved-caspase 3, LC3A/B, p-DRP1, DRP1 (Cell Signaling Technology), and *β*-Actin (ABclonal Biotechnology) and a horseradish peroxidase-conjugated secondary antibody (Bioyear Biotechnology) were used. ImageJ software was used to quantify the density of the blots.

### 2.12. Real-Time Polymerase Chain Reaction (RT-PCR)

The RNAiso Plus kit (Takara Bio, Kusatsu, Japan) was used to isolate total RNA. After evaluating the purity and measuring the concentration of total RNA, reverse transcription was conducted with the cDNA RT-PCR kit (Takara, Dalian, China). Subsequently, the ABI Fast 7900 HT real-time PCR system (Applied Biosystems, Foster City, CA, USA) was used for quantification. The primer sequences that were used in this study were presented as follows (synthesized by Sangon Biotech): human MTMR14 forward, 5′-GGCTCTAAGGTTGAGCGCAT-3′ and reverse, 5′-GAGCGTCCATACAGCTCTCC-3′; human *β*-Actin forward, 5′-GTCATTCCAAATATGAGATGCGT-3′ and reverse, 5′-GCTATCACCTCCCCTGTGTG-3′; mouse MTMR14 forward, 5′-AGACCTCATTCACCGAAGCA-3′ and reverse, 5′-TGTCACCACTCCGAAGAACA-3′; and mouse *β*-Actin forward, 5′-AACCCTAAGGCCAACCGTGAAA-3′ and reverse, 5′-GATGGCGTGAGGGAGAGCATA-3′.

### 2.13. Enzyme-Linked Immunosorbent Assay (ELISA)

Cell cultures and BALF supernatants in mice were first harvested and then centrifuged at 3,000 rpm for 5 minutes at 4°C. The levels of IL-6 and IL-8 in cell cultures were determined by the IL-6 (DY-206) and IL-8 (DY208) DuoSet ELISA kits (R&D Systems, USA). The concentration of KC (DY453) and IL-6 (DY406) in BALF supernatants was detected by ELISA kits (R&D Systems, USA) in accordance with the manufacturer's protocol.

### 2.14. Apoptosis Assay

The determination of apoptosis was performed using an Annexin V-APC apoptosis detection kit (BioLegend, USA). Following seeding of the cells in six-well plates, they were treated for 24 h with 5% CSE. The floating cells and adherent cells were transferred to tubes, washed two times with precooled PBS, and then resuspended in binding buffer. Next, cells were incubated in the dark at room temperature in the presence of Annexin V-APC and propidium iodide (PI) for 15 minutes. The proportion of apoptotic cells (Annexin V^+^/PI^−^ and Annexin V^+^/PI^+^) was then analyzed by flow cytometry (BD Biosciences).

### 2.15. Mitochondrial Reactive Oxygen Species (mtROS) Measurement

The level of mtROS was measured in cells by MitoSOX staining (Invitrogen), which is a live-cell permeant dye that selectively and rapidly targets mitochondria, following the manufacturers' instructions (for 10 minutes in the dark at 37°C). The fluorescence of MitoSOX Red was detected under a confocal microscope (Zeiss, Germany).

### 2.16. Detection of ROS

After treatments, DCFH-DA (Beyotime) working solution was added to the cells and then incubated for 30 min at 37°C. Flow cytometry (BD Biosciences) was used to measure the intensity of fluorescence. For mice, the *in situ* generation of superoxide anion in frozen and unfixed optimal cutting temperature (OCT) sections of lung tissue was evaluated by the oxidative fluorescent dye dihydroethidium (D7008, Sigma-Aldrich). DAPI was applied to counterstain nuclei. Slides were observed by fluorescence microscope, and images were collected.

### 2.17. Mitochondrial Transmembrane Potential Measurement

The levels of mitochondrial transmembrane potential (MMP) were detected by staining the treated cells with JC-1 fluorescence dye (Beyotime) based on the manufacturers' instructions. In healthy mitochondria, JC-1 forms aggregates that emit red fluorescence, while monomers emit green fluorescence in depolarized mitochondria. The intensity of fluorescence was detected under an inversion fluorescence microscope and flow cytometry (BD Biosciences). The results are stated as the percentage of green fluorescence intensity.

### 2.18. Statistical Analysis

Prism version 7.0 (GraphPad Software) was used for data analysis. Experimental data are stated as mean ± SEM. For comparisons between two groups, the Student *t*-test was performed, while one-way ANOVA was conducted for multiple comparisons. A *P* value of <0.05 was deemed as statistically significant.

## 3. Results

### 3.1. MTMR14 Was Downregulated in the Lungs of COPD Patients

**Figure 1:**
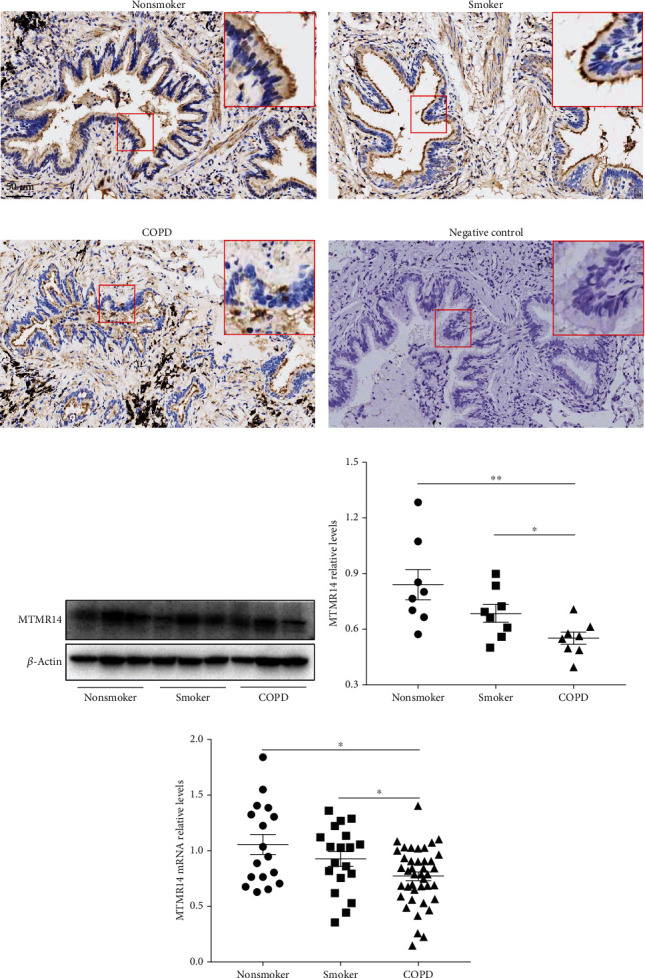
MTMR14 was downregulated in lungs of COPD patients. Representative immunohistochemical images of human lung tissue sections from nonsmokers (a), smokers (b), COPD patients (c), and negative control (d). Scale bars = 50 *μ*m, magnification = 200x. Western blot analysis of MTMR14 expression in whole-lung tissue homogenates (e). The band intensity was quantified by ImageJ (f), *n* = 8 per group. The relative mRNA expression of MTMR14 in lung tissues (g). Nonsmokers (*n* = 17), Smokers (*n* = 19), and COPD patients (*n* = 41). ^∗^*P* < 0.05 and ^∗∗^*P* < 0.01.

To identify the role of MTMR14 in COPD, we first compared the pulmonary expression of MTMR14 in nonsmokers, smokers, and COPD patients. The immunohistochemistry analysis revealed that the protein level of MTMR14 in the epithelium of COPD patients was markedly reduced compared with the other groups (Figures 1(a)–1(d)). Consistently, the Western blot analysis also showed significantly downregulated MTMR14 in the pulmonary tissue homogenates of COPD patients (Figures 1(e) and 1(f)). Lastly, we also detected the mRNA expression of MTMR14 in whole-lung tissue homogenates by RT-PCR. A total of 77 patients were recruited, including 41 COPD patients, 19 smokers without COPD, and 17 nonsmokers. These patients were matched for sex and age. The smoking index of non-COPD smokers and COPD patients, all current smokers, showed no significant differences. As expected, the FEV_1_, FEV_1_/FVC, and predicted FEV_1_% of COPD patients were significantly lower than those of non-COPD smokers and nonsmokers (Table 1). In addition, the COPD patients showed significantly decreased MTMR14 expression compared with nonsmokers and smokers (Figure 1(g)). The above findings suggest that MTMR14 is involved in COPD and may play a biological role in airway epithelium.

### 3.2. MTMR14 Was Downregulated in the Lungs of COPD Mice and CSE-Treated HBE Cells

**Figure 2:**
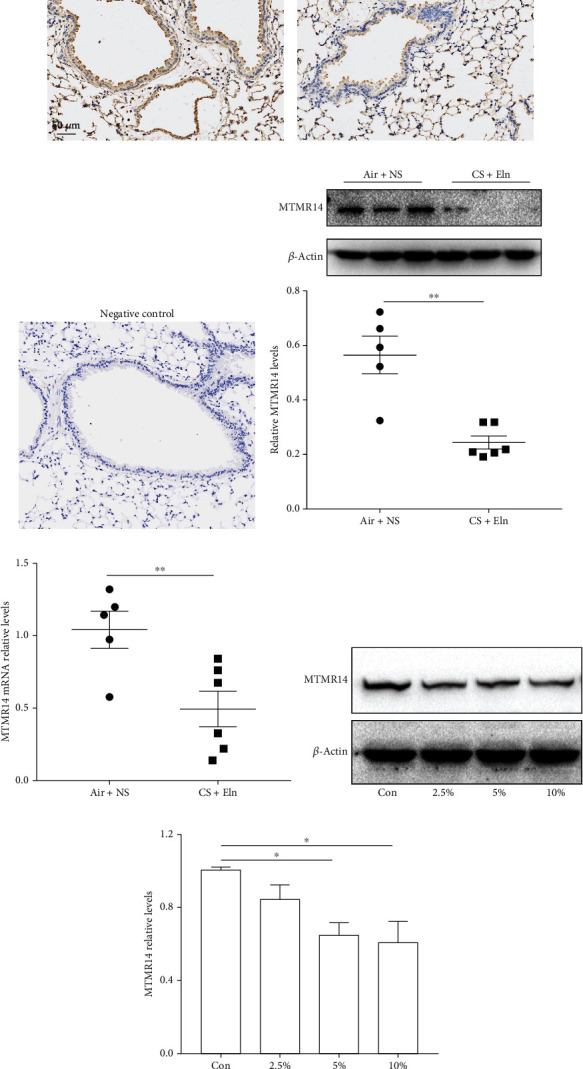
MTMR14 was downregulated in lungs of COPD mice and CSE-treated HBE cells. Representative immunohistochemical images of mouse lung tissue sections from the Air+NS (a), CS+Eln (b), and negative control (c) groups. Scale bars = 50 *μ*m, magnification = 200x. Western blot analysis and quantification of MTMR14 expression in whole-lung tissue homogenates (d). The relative mRNA expression of MTMR14 in mouse lung tissues (e). Every dot represents an independent mouse, Air+NS (*n* = 5) and CS+Eln (*n* = 6). Western blot and relative quantitative analysis of MTMR14 expression in HBE cells exposed to CSE at different concentrations (2.5%, 5%, and 10%) for 24 hours (f, g). ^∗^*P* < 0.05; ^∗∗^*P* < 0.01.

To further explore the biological role of MTMR14, we constructed a COPD mouse model. The results of immunohistochemical staining (Figures 2(a)–2(c)), Western blot (Figure 2(d)), and RT-PCR (Figure 2(e)) showed that MTMR14 was extensively expressed in the lung tissues of mice, in particular in the bronchial epithelium, while the expression of MTMR14 was significantly decreased in COPD mice. It is universally acknowledged that cigarette smoking is the primary risk factor for COPD. As the first-line defense against noxious insults, the bronchial epithelium has an indispensable role in preventing the onset of COPD. To further identify the function of MTMR14 in respiratory epithelial cells during COPD progression, we also investigated the influence of CSE on MTMR14 expression *in vitro* by incubating HBE cells with CSE in different concentrations (2.5%, 5%, and 10%) for 24 h. The results revealed that the protein level of MTMR14 began to significantly decline (Figures 2(f) and 2(g)).

### 3.3. MTMR14 Overexpression Suppressed CSE-Induced Inflammation and Apoptosis in HBE Cells

**Figure 3:**
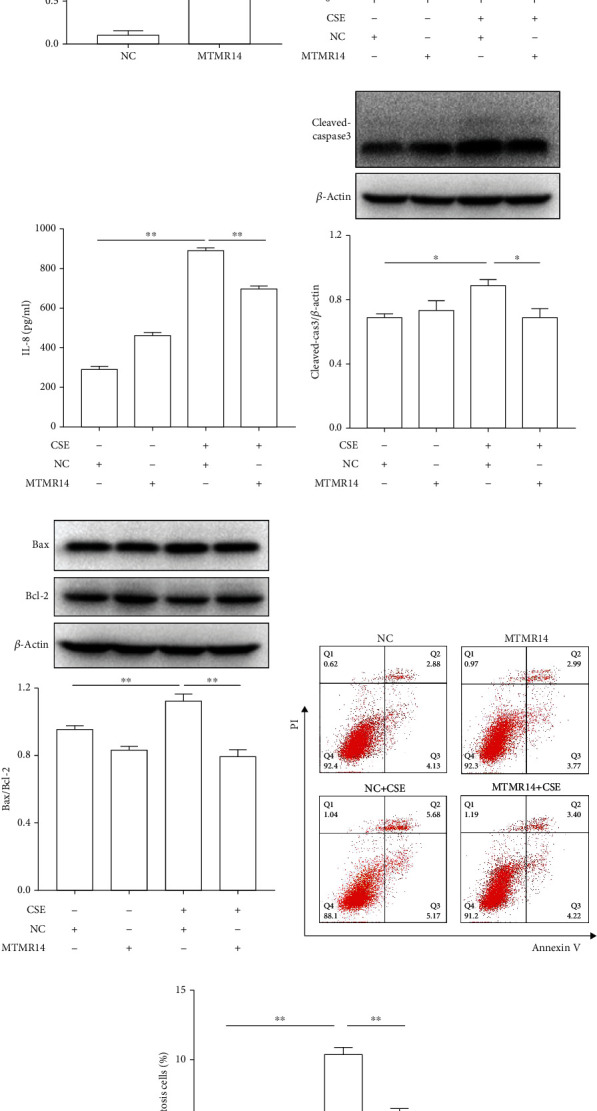
MTMR14 overexpression suppressed inflammation and apoptosis induced by CSE in HBE cells. Western blot was used to quantify the protein level of MTMR14 in HBE cells transfected with negative control lentiviral vectors (NC) or MTMR14-overexpressing lentiviral vectors (MTMR14) (a). CSE-induced upregulation of IL-6 and IL-8 in HBE cells was inhibited by the overexpression of MTMR14 (b, c). The levels of apoptosis-related proteins (Cleaved-caspase3, Bax, and Bcl-2) in HBE cells transfected with NC/MTMR14 and then treated with 5% CSE were measured and quantified (d, e). Flow cytometry was performed to detect apoptosis of treated HBE cells (f). The bar graph represents the percentage of apoptotic cells (g). Quantitative data are presented as mean ± SEM of three or more independent experiments. NC: negative control. ^∗^*P* < 0.05; ^∗∗^*P* < 0.01.

The effect of MTMR14 overexpression on CSE-treated HBE cells was evaluated to investigate whether MTMR14 had an impact on inflammation and apoptosis in airway epithelium. Overexpression of MTMR14 was effectively achieved by transfecting lentiviral vectors (Figure 3(a)). Then, we examined the levels of IL-6 and IL-8 24 h after treatment with 5% CSE. The results indicated that MTMR14 overexpression significantly inhibited the upregulation of inflammatory cytokines resulting from exposure to CSE (Figures 3(b) and 3(c)). Next, we analyzed the expression of apoptosis-related proteins in CSE-untreated and CSE-treated cells. The level of Cleaved-caspase 3 and the ratio of Bax/Bcl-2 in CSE-treated cells were noticeably increased, while MTMR14 overexpression inhibited CSE-enhanced apoptosis in HBE cells (Figures 3(d) and 3(e)). To further confirm that MTMR14 overexpression impeded cell apoptosis, we analyzed the proportion of apoptotic cells by flow cytometry. It was found that MTMR14 upregulation significantly inhibited CSE-induced apoptosis (Figures 3(f) and 3(g)). These results confirm that the overexpression of MTMR14 ameliorated the COPD-associated phenotype.

### 3.4. Overexpression of MTMR14 Ameliorates CSE-Induced Mitochondrial Damage and Excessive Mitophagy

**Figure 4:**
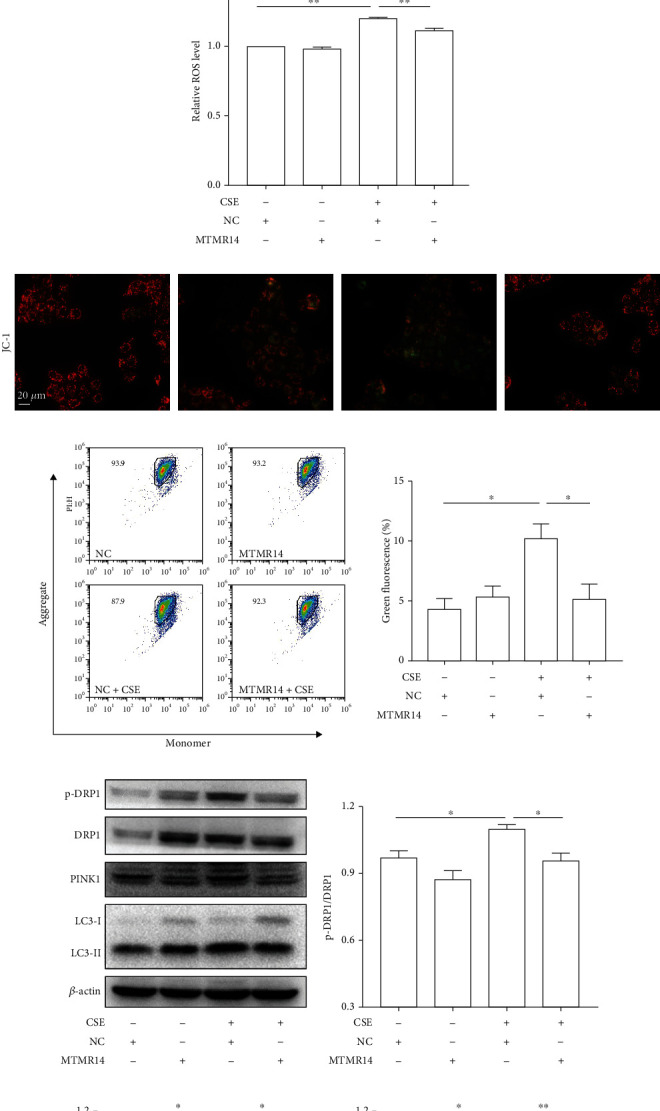
Overexpression of MTMR14 ameliorates CSE-induced mitochondrial damage and excessive mitophagy. Transmission electron microscope detection of mitochondria morphology and mitophagy vacuoles in HBE cells (a). HBE cells transfected with NC/MTMR14 treated with 5% CSE for 6 h; the black arrowheads represent mitochondria, and the red arrowheads indicate mitophagy vacuoles. Scale bar = 600 nm, magnification = 5000x. Photographs of fluorescence staining of untreated and treated HBE cells with MitoSOX Red (b) and intracellular ROS were determined by flow cytometry (c). HBE cells transfected with NC/MTMR14 treated with 5% CSE for 6 h; scale bar = 10 *μ*m. Following transfection of the HBE cells, they were stimulated with 5% CSE for 24 h, and MMP levels were analyzed under an inversion fluorescence microscope (d) and flow cytometry (e); scale bar = 20 *μ*m. Red fluorescence: healthy mitochondria; green fluorescence: depolarized mitochondria. Western blot analysis revealed that MTMR14 overexpression significantly suppressed the CSE-induced increased expression of mitophagy-related proteins (p-DRP1, PINK1, and LC3II/I) in the whole-cell lysates (f–i). Quantitative data are presented as mean ± SEM of three or more independent experiments. NC: negative control; MMP: mitochondrial membrane potential. ^∗^*P* < 0.05; ^∗∗^*P* < 0.01.

Mitochondrial homeostasis, which is regulated by mitochondrial division and mitophagy synergistically, is an essential condition for maintaining mitochondrial function and ensuring cell function. Mitochondrial function and mitophagy were detected to elucidate the possible mechanisms by which MTMR14 overexpression regulates the biological behavior of HBE cells. The TEM confirmed that restored MTMR14 ameliorated CSE-induced mitochondrial crest breaks and morphological damage, while the formation of mitochondrial autophagic structures and less damaged mitochondria encapsulated by a bilayer membrane were detected (Figure 4(a)). Consistently, the abnormal increase in mitochondrial and intracellular ROS (Figures 4(b) and 4(c)) and the decrease in MMP (Figures 4(d) and 4(e)) induced by CSE were improved. Furthermore, the results of Western blot analysis suggested that MTMR14 overexpression decreased the elevation of the ratio of p-DRP1/DRP1, PINK1/*β*-Actin, and LC3 II/I induced by CSE (Figures 4(f)–4(i)). To sum up, overexpressing of MTMR14 ameliorates CSE-induced mitochondrial damage and excessive mitophagy, which may be one of the mechanisms to regulate inflammation and apoptosis.

### 3.5. Overexpression of MTMR14 Inhibits the Progression of COPD in Mice

**Figure 5:**
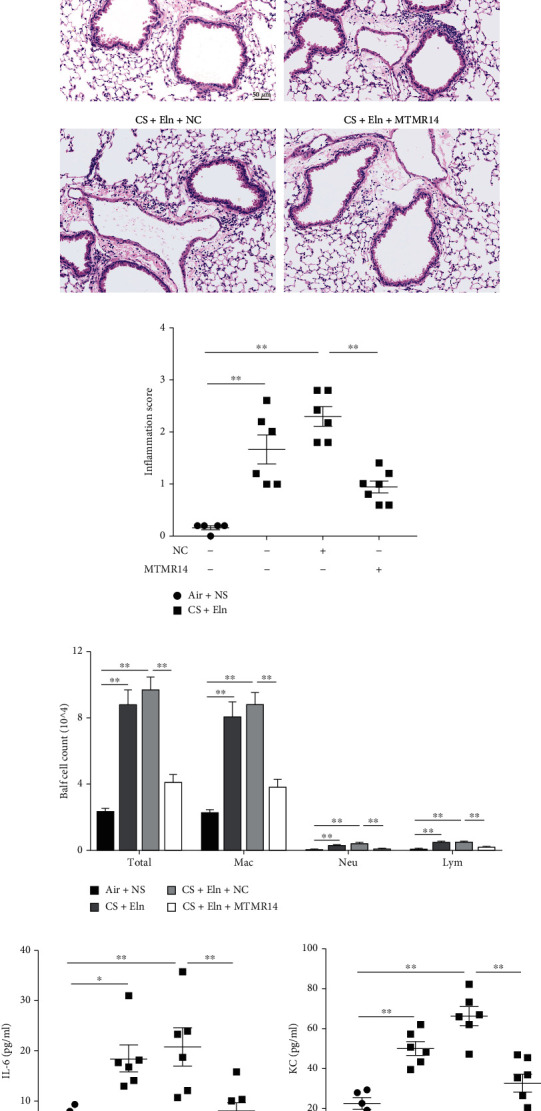
MTMR14 overexpression inhibited inflammation in COPD model mice. Experimental schedule (a). The representative images of HE-stained lung paraffin sections indicate inflammatory cell infiltration (b) Scale bars = 50 *μ*m, magnification = 200x. Semiquantitative score of inflammatory cell infiltration in mice (c). Number of total cells, macrophages, neutrophils, and lymphocytes in BALF (d). Levels of IL-6 and KC in BALF supernatant determined by ELISA (e, f). *n* = 5-7 mice per group. NC: negative control. The data are presented as mean ± SEM, and every dot represents an independent mouse. ^∗^*P* < 0.05; ^∗∗^*P* < 0.01.

**Figure 6:**
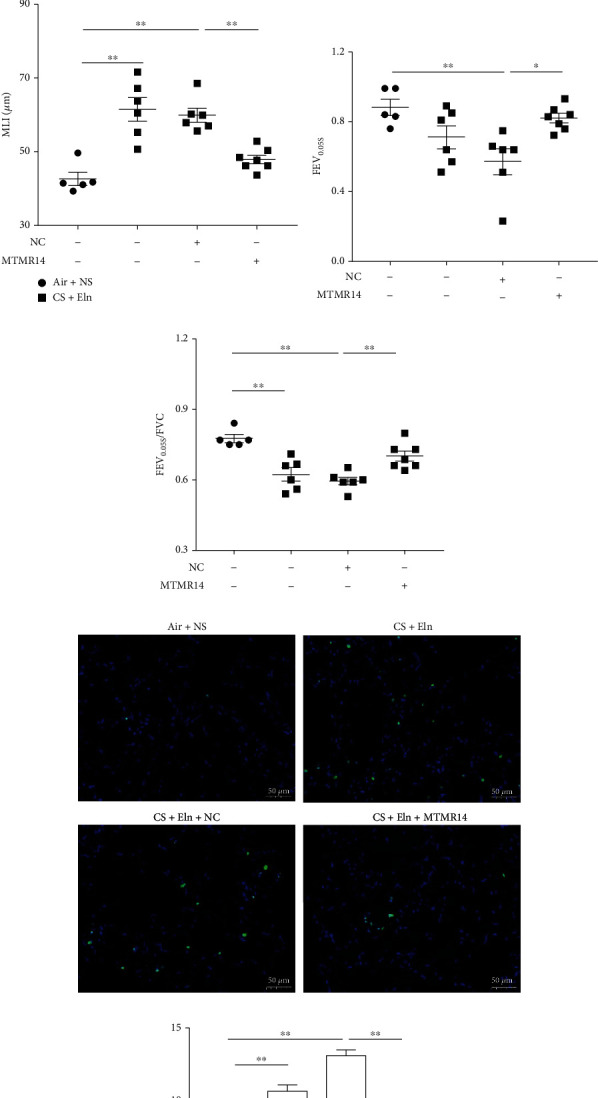
MTMR14 overexpression prevented emphysema and improved lung function in COPD model mice. The representative images of HE-stained lung paraffin sections indicate the pathological conditions of mice (a). The mean linear intercept (MLI) was calculated to evaluate the spatial size (b). The results of FEV_0.05s_ and FEV_0.05s_/FVC (c, d), *n* = 5-7 mice per group. Apoptosis detected by TUNEL staining (e, f). Scale bars = 50 *μ*m, magnification = 200x; nuclear stain DAPI: blue; TUNEL staining: green. *n* = 3 mice per group. NC: negative control; FEV_0.05s_: forced expiratory volume in 0.05 seconds; FVC: forced vital capacity. The data are presented as mean ± SEM, and every dot represents an independent mouse. ^∗^*P* < 0.05; ^∗∗^*P* < 0.01.

To further demonstrate the role of MTMR14 in COPD, we successfully constructed a COPD mouse model with MTMR14 overexpression (Figure 5(a)). We detected green fluorescence in lung tissue to evaluate the successful infection of AAVs (Supplementary Figure 1) and assessed inflammation in the aspects of HE staining, BALF count, and ELISA. The results have shown that the infiltration of peribronchial inflammatory cells, as well as the inflammatory score, was both significantly decreased in the CS+Eln+MTMR14 group compared with the CS+Eln+NC group (Figures 5(b) and 5(c)). According to the BALF cell count, the total number of BALF cells, macrophages, and neutrophils in the CS+Eln+MTMR14 group was significantly reduced in comparison to that of the CS+Eln+NC group (Figure 5(d)). In addition, the levels of IL-6 and KC in BALF supernatants were measured and consistent with the results above (Figures 5(e) and 5(f)). More importantly, MTMR14 overexpression significantly improved the destruction of alveolar structures (Figures 6(a) and 6(b)) and the decline of lung function in CS+Eln and CS+Eln+NC mice (Figures 6(c) and 6(d)). Furthermore, TUNEL staining was used to detect apoptosis, and the results indicated that apoptosis was increased in CS+Eln and CS+Eln+NC mice but reduced in CS+Eln mice after the delivery of overexpressed MTMR14 (Figures 6(e) and 6(f)). These findings indicate that MTMR14 overexpression can suppress COPD progression in mice.

### 3.6. Overexpression of MTMR14 Partially Inhibits Mitochondrial Damage in Mice

**Figure 7:**
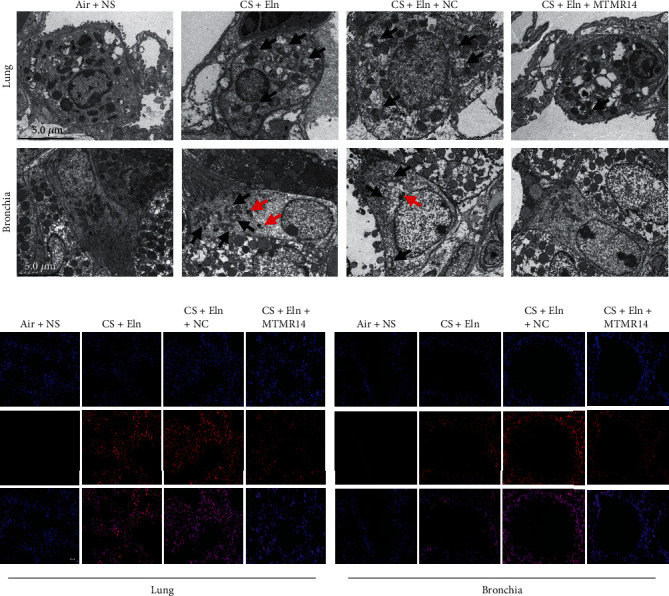
Overexpression of MTMR14 partially inhibits mitochondrial damage in mice. Transmission electron microscope detection of mitochondria morphology and mitophagy in the bronchial epithelium as well as lung parenchyma cells in mice (a). The black arrowheads represent damaged mitochondria, and the red arrowheads indicate mitophagy vacuoles; Scale bar = 5.0 *μ*m. The oxidative fluorescent dye dihydroethidium was applied to assess the in situ production of superoxide anions in frozen and unfixed lung tissue OCT sections. Photographs of fluorescence staining (b). Scale bars = 50 *μ*m, magnification = 200x.

To further verify that MTMR14 overexpression improves mitophagy and mitochondrial function to a certain extent *in vivo*, we first applied TEM to observe the mitochondrial structure and mitophagy. As shown in Figure 7(a), MTMR14 overexpression inhibited CS+Eln-induced mitochondrial crest destruction and the increase of the number of swollen mitochondria in the bronchial epithelium as well as lung parenchyma cells. Mitochondria are both the primary producers of oxygen radicals and the target of their destruction. The oxidative fluorescent dye dihydroethidium was applied to assess the *in situ* production of superoxide anions in frozen and unfixed lung tissue OCT sections. The results showed that MTMR14 overexpression inhibited the CS+Eln-induced increase of ROS in the bronchial epithelium as well as lung parenchyma cells (Figure 7(b)).

## 4. Discussion

The main results of this study can be summarized as follows: (1) the significant lower expression of MTMR14 in the lung of COPD patients compared with controls indicates the possibility of MTMR14 as a novel regulatory mechanism; (2) in *vitro*, MTMR14 inhibited CSE-induced inflammation and apoptosis in bronchial epithelial cells, and further mechanism studies revealed that the biological regulation of MTMR14 was, to a certain extent, dependent on the regulation of mitochondrial function and mitophagy; and (3) in *vivo*, MTMR14 reduced inflammation and improved emphysema in COPD model mice. Consistent with the results in *vitro*, MTMR14 overexpression inhibited the excessive mitophagy, alleviated mitochondrial damage, and decreased the production of ROS in COPD model mice.

Human bronchial epithelial cells are the site of first contact for environmental stimuli and perform a pivotal role in maintaining airway function. Studies on bronchial biopsies of COPD patients reported increased expression of inflammatory genes and proteins, as well as structural alterations in bronchial epithelial cells [1]. CS is the main etiological factor of COPD. Upregulation of inflammatory cytokines (e.g., IL-6 and IL-8) induced by CS treatment may be correlated with pulmonary function, disease severity, and clinical outcomes of COPD patients [20, 21]. Apoptosis, a process of orderly and spontaneous cell death controlled by genes, plays an important role in eliminating overactive inflammatory cells. However, in COPD patients, alveolar macrophages are less effective in the phagocytosis of apoptotic airway epithelial cells, which puts pressure on maintaining a stable internal environment [22]. Apoptosis has been currently recognized as the fourth pathogenic cause of COPD [4, 5, 23, 24]. Excessive apoptosis may be counterproductive, resulting in impaired host defenses. Thus, seemingly independent inflammatory responses and apoptosis are closely related, and their interaction may further aggravate COPD.

Recent studies have reported on the involvement of MTMR14 in human disease. Lv et al. [12] showed that MTMR14 deficiency led to late-onset inflammation. Consistent with this, mice lacking MTMR14 showed accelerated lipid accumulation and inflammation induced by high-fat diet [13]. Furthermore, it was also reported that MTMR14 knockdown promoted apoptosis in liver cancer cells [14]. Thus, we hypothesized that MTMR14, a molecule proven to be involved in inflammation and apoptosis, might be a new target for COPD. We first found that the expression of MTMR14 was downregulated in COPD patients and COPD model mice, as well as CSE-stimulated HBE cells. The *in vitro* study further revealed that MTMR14 overexpression partially suppressed CSE-induced inflammation and apoptosis. Finally, *in vivo* studies confirmed that MTMR14 inhibited inflammation and the development of emphysema in COPD model mice. These findings imply that MTMR14 plays a critical role in COPD by regulating inflammation and apoptosis.

Mitochondria are the key cellular source of reactive species and the powerhouse of cells capable of generating energy. Mitochondrial homeostasis and mitochondrial dysfunction are sustained by an integrated mitochondrial structure, balanced mitochondrial calcium, stabilization of the mitochondrial membrane potential, and prompt removal of damaged mitochondria through mitophagy [25]. The dysfunction of mitochondria plays a crucial role in modulating inflammation, oxidative stress, and apoptosis, which are related to the pathogenesis of chronic respiratory diseases, for instance, pulmonary fibrosis and COPD [26, 27]. Accumulating evidence has indicated that mitochondrial dysfunction in COPD is mainly manifested in the increase of ROS, reduction of the mitochondrial membrane potential, destruction of mitochondrial structure, and mitophagy imbalance [28–31]. So far, studies on mitophagy in COPD pathogenesis have been highly controversial. Mizumura et al. reported that inhibition of mitophagy prevents CS-induced mitochondrial dysfunction, mucociliary clearance, and airspace enlargement in mice [32]. Similarly, Nix/FUNDC1 overexpression further activated CSE-induced mitophagy, leading to aggravated inflammation and cell injury and the promotion of COPD progression [33, 34]. Consistent with these studies, our results suggest that the ameliorative effect of MTMR14 on the progression of COPD may also be achieved by inhibiting excessive mitophagy and improving mitochondrial function. Contradictorily, other researches have indicated that activating mitophagy can improve mitochondrial dysfunction, retard cellular senescence, and ultimately inhibit pathological COPD progression [29, 35, 36]. These findings once again show that activated mitophagy has a dual role in COPD, which is usually associated with a specific scenario [25]. However, it is undeniable that mitophagy is involved in a variety of processes, and the maintenance of mitophagy homeostasis is essential to ensure mitochondrial function and regulate the disease process.

There are some limitations in this study that should be addressed. Firstly, the exploration of this mechanism is still in the preliminary stage. On the one hand, mitophagy induced by damage in COPD is mainly caused by two major proteins, PINK1, the serine/threonine protein kinase encoding PTEN-induced putative kinase, and an E3 ubiquitin protein ligase known as Parkin (PARK2) [37, 38]. Mizumura et al. have shown that lung epithelial cells, which were obtained from COPD patients, displayed an enhanced expression of PINK1. Genetic deficiency of PINK1 protects against CS-induced necroptosis and mitochondrial dysfunction *in vitro* [32]. Conversely, Ito *et al.* showed that protein levels of Parkin were reduced in the lungs of COPD patients [35]. Reduced Parkin levels accelerate cellular senescence *in vivo* and *in vitro* [35, 36]. In this study, our results suggest that MTMR14 inhibits the CS-induced elevation of PINK1. However, due to the low expression of Parkin in epithelial cells, the regulatory effect of MTMR14 on Parkin is currently unknown (relevant data are not shown). Dynamin-related protein 1 (DRP1) has been identified as a regulator of mitochondrial fission [39]. In addition, the triggering effect of DRP1 on mitophagy has also received increased attention and knockdown of DRP1 or Mdivi-1 (a mitochondrial fission/mitophagy inhibitor) treatment inhibits disease progression [32, 40, 41]. Unfortunately, we have not verified the specific molecular targets by which MTMR14 plays its biological role in the regulation of mitophagy. On the other hand, a summary of relevant studies suggests that the mechanism by which MTMR14 regulates the disease process is mainly the Akt signaling pathway. Zhang *et al.* reported that the prevention of cardiac hypertrophy by MTMR14 involves the elevation of Akt pathway components [42]. In addition, Li *et al.* showed that MTMR14 plays a protective role in hepatic ischemia-reperfusion injury by interacting with the Akt signaling pathway [43]. The Akt signaling pathway is also a key component in the regulation of inflammation and emphysema in COPD [44, 45]. Whether the regulatory role of MTMR14 in COPD is partly due to Akt signaling remains unexplored. Secondly, knockdown of MTMR14 *in vivo* and *in vitro* to further verify its biological effect on COPD is also significant and necessary. All aforementioned will be the focus of our follow-up study.

## 5. Conclusion

In conclusion, our results provide the first evidence of the regulatory role of MTMR14 in COPD. MTMR14 alleviates inflammation and emphysema in COPD, which depends, to a certain extent, on the regulation of mitochondrial function and mitophagy. Our study supports the development of novel treatment strategies for COPD patients that target MTMR14. The clinical value and underlying mechanisms of MTMR14 in COPD development are worthy of further investigation.

## Figures and Tables

**Table 1:** Clinical characteristics of patients who supply lung tissue.

	Nonsmoker	Smoker	COPD
Subjects	17	19	41
Sex (M/F)	17/0	19/0	41/0
Age (years)	58.8 ± 0.9	60.7 ± 2.0	60.6 ± 0.9
Smoking index (p.y)	0	33.0 ± 5.4	40.7 ± 2.5
FEV_1_ (L)	3.05 ± 0.07^∗∗^	2.93 ± 0.11^∗∗^	2.33 ± 0.08
FEV_1_%pred	104.10 ± 2.53^∗∗^	106.70 ± 4.06^∗∗^	79.78 ± 2.47
FVC (L)	3.94 ± 0.12	3.83 ± 0.15	3.79 ± 0.10
FEV_1_/FVC (%)	77.81 ± 1.30^∗∗^	76.40 ± 0.83^∗∗^	61.19 ± 1.11

Values are presented as mean ± SEM. COPD: chronic obstructive pulmonary disease; FVC: forced vital capacity; M/F: male/female; p.y: pack-years; FEV_1_: forced expiratory volume in one second; %pred: %predicted; ∗∗: *P* < 0.01 versus the COPD group.

## Data Availability

All data used to support the findings of this study are included within the article. Raw data used to generate the figures are available from the corresponding author upon request.

## Supplementary Materials

Supplementary Materials Supplemental Figure 1: the AAV infected the lung tissues of mice. The green fluorescent signal indicated AAV-positive cells in the three mouse groups: Negative control, NC-AAV, or MTMR14-AAV intratracheally administrated.
