# Supplementary material for: MTMR14 Alleviates Chronic Obstructive Pulmonary Disease as a Regulator in Inflammation and Emphysema
Source: Oxid Med Cell Longev. 2022 Jan 7;2022:9300269. doi: 10.1155/2022/9300269 (PMC8759842; doi:10.1155/2022/9300269)

**MTMR14 alleviates chronic obstructive pulmonary disease as a regulator in inflammation and** **emphysema**

Yiya Gu^1^, Jinkun Chen^2^, Qian Huang^1^, Yuan Zhan^1^, Ting Wang^1^, Jixing Wu^1^, Jianping Zhao^1^, Zhilin Zeng^3^, Yongman Lv^4^, Chengfeng Xiao^5*^, Jungang Xie^1*^

**Supplemental Figure 1. The AAV infected the lung tissues of mice**

The green fluorescent signal indicated AAV-positive cells in the three mice groups: Negative Control, NC-AAV or MTMR14-AAV intratracheally administrated.


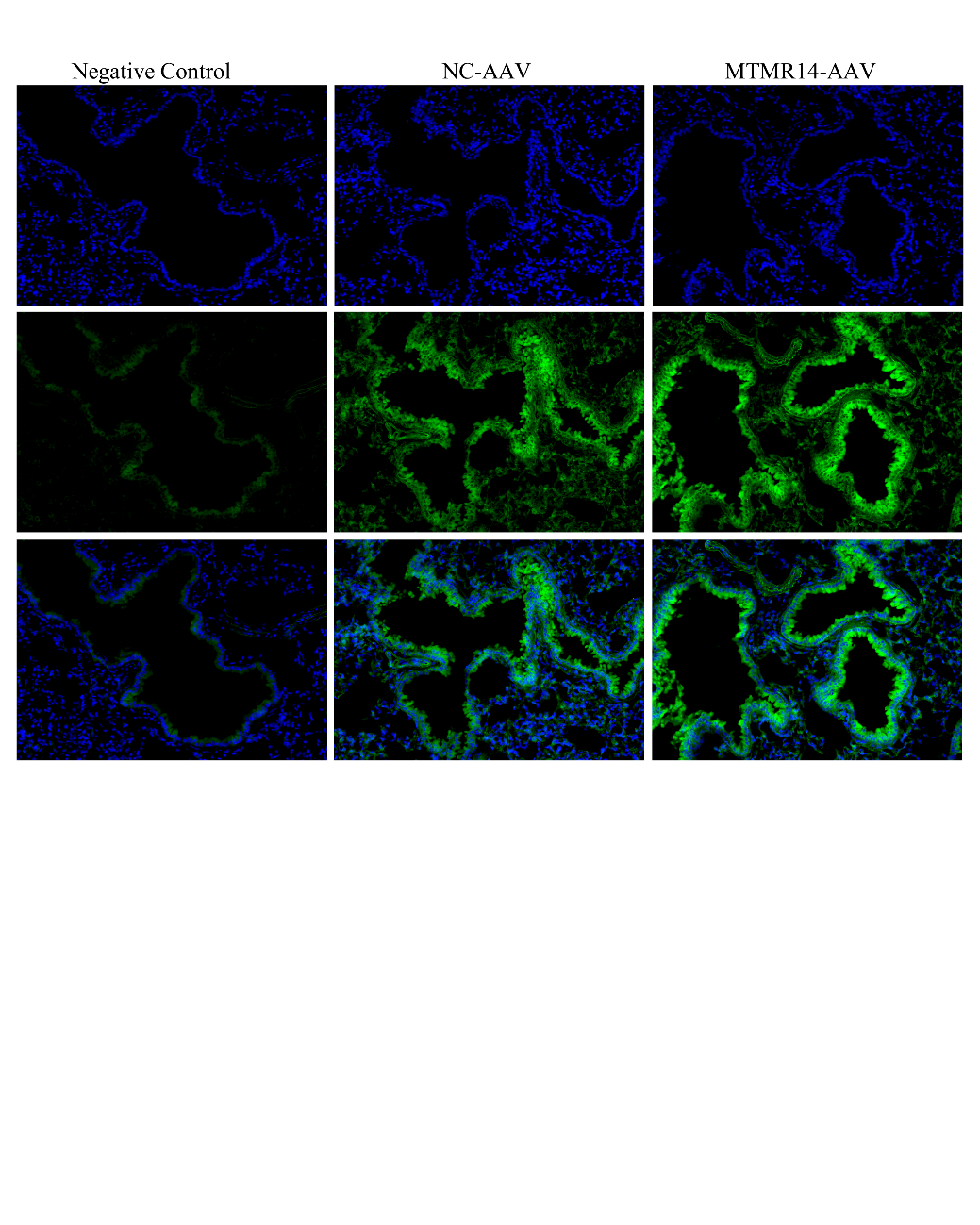

Supplement: Supplementary Materials — Supplemental Figure 1: the AAV infected the lung tissues of mice. The green fluorescent signal indicated AAV-positive cells in the three mouse groups: Negative control, NC-AAV, or MTMR14-AAV intratracheally administrated. [file 9300269.f1.zip › Supplemental Files (1).docx]
